# Structural and Organizational Dimensions of Compassion Fatigue in Pediatric Oncology Nursing: A Qualitative Study

**DOI:** 10.3390/ijerph23080981

**Published:** 2026-07-28

**Authors:** Teresa Galanti, Morena Santoriello, Michela Cortini, Elisa Di Tullio, Angelica Di Febbo, Stefania Fantinelli, Gabriella Mincione

**Affiliations:** 1 Department of Psychology, “G. d’Annunzio” University of Chieti-Pescara, 66100 Chieti, Italy; 2 ASL Pescara, 65125 Pescara, Italy; 3 School of Medicine and Health Sciences, “G. d’Annunzio” University of Chieti-Pescara, 66100 Chieti, Italy; 4 Department of Theoretical and Applied Sciences, Center for Research in Applied Psychology, eCampus University, 00182 Rome, Italy; 5 Department of Innovative Technologies in Medicine & Dentistry, “G. d’Annunzio” University of Chieti-Pescara, 66100 Chieti, Italy

**Keywords:** compassion fatigue, healthcare psychosocial risk, workplace health promotion, evolving workplaces, qualitative descriptive design, computer-assisted lexical analysis

## Abstract

**Highlights:**

**Public health relevance—How does this work relate to a public health issue?**

- Pediatric oncology nursing is a high-intensity care specialty in which repeated exposure to child death and sustained emotional investment in patient–family relationships constitute a recognized occupational psychosocial risk, with direct implications for workforce sustainability and health system capacity.
- In Italy, structural conditions—including the virtual absence of a national pediatric hospice network and a fragmented regulatory framework for end-of-life education—compound this risk and remain systematically unaddressed by existing public health workforce policies.

**Public health significance—Why is this work of significance to public health?**

- To the best of the authors’ knowledge, this study provides novel qualitative evidence, supported by computer-assisted textual analysis, that compassion fatigue-related experiences and reported intention to leave among Italian pediatric oncology nurses are perceived by participants as structurally generated rather than as individual clinical vulnerabilities, with implications for SDG 3 (Good Health and Well-Being) and SDG 8 (Decent Work and Economic Growth).
- The analysis reveals that nurses in this setting lack any organizational vocabulary for peer-level or team-level support, indicating that a culture of collective professional sustainability has not been institutionally cultivated—a gap with direct consequences for care quality and patient safety.

**Public health implications—What are the key implications or messages for practitioners, policy makers and/or researchers in public health?**

- Dedicated end-of-life curricula, structured psychological debriefing protocols, and formalized peer support systems must be institutionalized as organizational standards in pediatric oncology settings, not treated as optional professional development add-ons.
- National health workforce planning frameworks should mandate multi-level sustainability strategies—integrating educational preparation, organizational support structures, and pediatric hospice infrastructure investment—as prerequisites for sustainable specialized nursing practice.

**Abstract:**

Pediatric oncology nursing is among the highest-intensity care specialties, exposing nurses to repeated child death and sustained emotional investment in family relationships. This occupational burden is a recognized psychosocial risk factor and a driver of compassion fatigue, burnout, and reported intention to leave, yet the organizational and educational determinants of this risk remain poorly captured by existing assessment approaches. This study investigated the lived experiences of nurses caring for terminally ill children in a pediatric onco-hematology unit and hospice ward in central Italy (N = 15), using a qualitative descriptive design, informed by a phenomenological sensibility to lived experience, supported by computer-assisted textual analysis. Structured face-to-face interviews were analyzed through thematic content analysis with stepwise replication, while quantitative textual analysis was performed using T-Lab software to map co-occurrence patterns among key lemmas, providing a frequency-based, semantically grounded picture of nurses’ representations of occupational risk. Seven themes emerged, including quality of nursing care, parental relationships, coping with a child’s death, and impact on personal and professional life. Findings showed that reported intention to leave was associated, in participants’ accounts, with absent psychological debriefing, cumulative emotional burden, inadequate end-of-life training, and a near-total absence of organizational vocabulary for peer-level support—a gap directly visible in the co-occurrence structure of nurses’ language. As a secondary aim, by combining qualitative depth with complementary lexical analysis of thematic patterns, this study also offers a methodological example of how psychosocial risk in high-intensity care settings can be assessed and translated into actionable indicators for organizational climate and workforce well-being. The findings inform concrete prevention strategies and policy recommendations for nursing education and occupational health, consistent with this Special Issue’s focus on methodological innovation for psychosocial risk assessment and public health policy design.

## 1. Introduction

Psychological well-being among healthcare workers is a recognized correlate of care quality, patient safety, and the long-term viability of specialized health services [1]. In high-intensity care specialties, the conditions of work generate distinctive psychosocial risks that—when unaddressed by organizational and educational structures—translate into compassion fatigue (CF), burnout, and reported intention to leave. Despite growing recognition of these risks in occupational health research, their organizational and educational determinants in the most demanding clinical settings remain poorly characterized and systematically under addressed, particularly in contexts undergoing rapid institutional transformation.

Pediatric oncology nursing represents one of the highest-intensity care specialties in the healthcare system. Globally, more than 20 million children per year are eligible for pediatric palliative care (PPC) [1]; in Italy, over 11,000 children with incurable or terminal illness require PPC annually [2]. Oncological and hematological diseases represent the primary diagnosis in this population, making pediatric onco-hematology units and hospice wards the principal clinical contexts in which nurses confront end-of-life (EOL) care on a daily basis. The relational demands of this specialty—sustained emotional investment in dying children and their families, repeated bereavement, and the management of parental grief—constitute a specific and severe psychosocial risk exposure that is structurally inherent to the role, not reducible to individual clinical vulnerability.

This psychosocial risk exposure is best understood within the Job Demands–Resources (JD–R) framework [3,4], which conceptualizes occupational well-being as the outcome of the balance between job demands and job resources. Within a JD–R perspective, CF and burnout are not individual pathologies but indicators of a systematic deficit in job resources relative to job demands.

These psychosocial risks are further amplified by the evolving institutional context in which Italian pediatric oncology nurses operate. The 2016 Conferenza Stato–Regioni agreement on pediatric palliative care [5] formalized a structural transition from hospital-centered to home-based and hospice models of care, placing nurses at the intersection of organizational transformation, resource uncertainty, and expanding relational demands. This evolving workplace context—characterized by shifting care delivery models, ill-defined nursing roles, and underdeveloped palliative infrastructure—constitutes an additional and underexplored layer of psychosocial risk that compounds the individual and relational stressors already documented in this specialty, and that calls for a multi-level analytical approach capable of capturing both organizational conditions and their effects on workers’ psychological well-being.

Despite this evidence, the organizational and educational conditions that generate and sustain psychosocial risk in pediatric oncology nursing remain poorly understood and institutionally unaddressed, requiring a shift from individual-level clinical responses to organizational and policy-level investment in job resources. Within this framework, recurrent exposure to child death, sustained empathic engagement with families in crisis, and time pressure in under-resourced settings are classified as job demands; end-of-life training, structured psychological debriefing, and peer/team support are classified as job resources; and emotional exhaustion, compassion fatigue-related experiences, and reported intention to leave are classified as potential outcome variables.

This study’s primary aim was to investigate the psychosocial risk factors and psychological well-being correlates among nurses working in Italian pediatric onco-hematology settings, using a qualitative design supported by computer-assisted textual analysis. As a secondary, methodological aim, by mapping both the experiential and the semantic-structural dimensions of occupational risk in this specialty, the study also offers a methodological example of how psychosocial risk assessment can be applied to an evolving, high-intensity care context to generate actionable evidence for organizational climate improvement, workforce well-being promotion, and public health policy design.

## 2. Literature Review

The literature reviewed in this section is organized primarily around the Job Demands–Resources (JD–R) model which provides the analytical lens for understanding how organizational conditions generate or buffer psychosocial risk. The dual-process model of occupational well-being [3,4], understood here as an extension of JD–R that specifies two parallel pathways—an energetic/health-impairment pathway and a motivational pathway—is used to integrate risk prevention with the active promotion of psychological well-being, rather than as a competing framework. This dual framework is informed by the WHO Global Strategy on Human Resources for Health [6] and the Sustainable Development Goals, particularly SDG 3 and SDG 8. Within it, compassion fatigue, burnout, educational deficits, and institutional abandonment are conceptualized not as individual clinical phenomena but as systemic indicators of a structural imbalance between job demands and job resources [7,8,9,10]—measurable signals that the organizational conditions required for psychological well-being and sustainable specialized practice are not being met. Although related, these constructs are conceptually distinct. Compassion fatigue (CF) refers specifically to the progressive erosion of empathic capacity resulting from sustained engagement with others’ suffering; burnout is a broader syndrome of emotional exhaustion, cynicism, and reduced sense of accomplishment that can arise independently of empathic engagement; secondary traumatic stress denotes trauma-like symptoms arising from indirect exposure to another’s traumatic experience; moral distress refers to the psychological strain of knowing the ethically right course of action while being institutionally prevented from taking it; and compassion satisfaction denotes the positive counterpart to CF, reflecting the sense of meaning derived from caring effectively for others. CF and burnout, in particular, are not interchangeable: a nurse can be burned out without exhibiting the empathy-specific erosion characteristic of CF, and vice versa.

### 2.1. Compassion Fatigue and Burnout as Psychosocial Risk Outcomes

Compassion fatigue and burnout in pediatric oncology nursing are not episodic or individually determined phenomena: they are the predictable psychosocial outcomes of a chronic structural imbalance between exceptionally high job demands and systematically deficient job resources [3]. CF—defined as the progressive erosion of empathic capacity resulting from sustained engagement with others’ suffering [11]—represents the endpoint of a depletion trajectory that is occupationally produced and organizationally modifiable. Up to 60% of oncology nursing staff experience some form of burnout [9], with pediatric oncology nurses showing significantly higher levels of secondary traumatic stress than other nursing specialties due to the intensity of emotional bonds formed with child patients and their families [10].

The scale of this problem has been quantified at the workforce level using methodologically distinct approaches that nonetheless converge on the same pattern:: a large-scale survey of 6590 United States pediatric nurses found that close to 31% experienced CF and burnout often or daily, with years of experience inversely correlated with CF up to a threshold of 16 years, suggesting a form of survivor selection rather than genuine recovery [12]; a smaller study using validated psychometric instrument (Copenhagen Burnout Inventory; Kessler Psychological Distress Scale) on 188 pediatric oncology nurses similarly found client-related burnout, driven by repeated patient loss, to be the strongest predictor of psychological distress [9], lending psychometric confirmation to the pattern observed at scale in the larger survey.

It should be noted that individual factors—resilience, coping repertoire, and prior clinical experience—moderate, but do not eliminate, this structural imbalance. Evidence shows that even highly resilient nurses remain vulnerable to compassion fatigue when institutional job resources are absent, indicating that individual and organizational levels interact rather than substitute for one another, and that resilience-building alone cannot compensate for a structural deficit in job resources [13,14].

Beyond individual impact, burnout in this population has direct patient safety implications, being significantly associated with lower patient satisfaction and higher rates of adverse events in a meta-analysis of 85 studies and 288,581 nurses [15].

### 2.2. Psychological Well-Being as a Positive Construct: Beyond the Deficit Model

While CF and burnout represent the negative pole of the occupational health continuum, the call to promote psychological well-being in healthcare workplaces requires explicit attention to its positive dimensions. Ryff’s multidimensional model of eudaimonic well-being [16]—encompassing autonomy, personal growth, purpose in life, positive relations with others, environmental mastery, and self-acceptance—provides a conceptual framework for understanding how nurses in high-demand settings can sustain functional and meaningful practice despite chronic occupational stress. Within this framework, CF and burnout are not merely risk indicators: they represent deficits in specific well-being dimensions, most notably purpose in life and positive relations with others, whose erosion precedes and predicts workforce attrition. The Compassion Satisfaction dimension of the Professional Quality of Life Scale (ProQOL) [17] operationalizes this positive pole empirically: it captures the sense of meaning and fulfillment derived from caring effectively for others, which serves as a psychological resource that buffers against CF accumulation. Research on oncology nurses documents that compassion satisfaction is inversely correlated with burnout and CF [18], and that organizational conditions—including peer support, debriefing, and recognition of relational labor—are the primary determinants of whether compassion satisfaction is sustained or depleted over a nursing career [19]. Compassion satisfaction, emotional labor, and moral distress are treated in this study as auxiliary interpretive lenses, used to interpret specific facets of nurses’ accounts, rather than as parallel theoretical frameworks equivalent to JD–R; Ryff’s model, in particular, is applied as a post hoc interpretative lens for the positive well-being dimensions emerging from the data, rather than as a construct built into the interview guide or coding scheme; this dual-process perspective—integrating well-being promotion alongside JD–R as the primary risk prevention framework—is the conceptual orientation of the present study.

### 2.3. Educational Deficits: The Training Gap as a Psychosocial Risk Amplifier

The most consistently documented structural driver of psychosocial risk in pediatric EOL nursing is the systematic absence of formal preparation. Without structured pathways for developing EOL competence, each generation of nurses enters the specialty underprepared and absorbs occupational costs through trial-and-error learning—a pattern that functions as a chronic job resource deficit [3,4], amplifying emotional demands while leaving nurses without the educational tools required to manage them [20]. Across heterogeneous contexts—Turkish clinical settings [19], United States pediatric oncology units [21,22], and Korean oncology wards [23]—a convergent pattern emerges: EOL communicative competence is acquired informally through prolonged exposure rather than structured education, and is systematically underdeveloped in novice nurses; where structured education has been tested experimentally, it reliably improves self-rated competence and reduces distress, suggesting the deficit is remediable rather than intrinsic to the specialty.

### 2.4. Relational Dimensions of EOL Care: Family Centered Practice and Its Occupational Costs

EOL nursing in pediatric oncology is defined by its relational intensity, though the literature approaches this relational dimension from two distinct angles. One line of work frames family-centered engagement primarily as a communication and training challenge: Ruhe et al. [24] argue that including children in EOL discussions is an ethical imperative requiring age-adapted communication skills, and Ranallo [25] similarly reframes delayed integration of palliative care in pediatric oncology—often driven by families’ and providers’ reluctance to engage with it early—as an educational and communication barrier rather than an insurmountable clinical problem. A second line of work, in contrast, documents the occupational cost of this same relational positioning: Akard et al. [26,27] show that nurses’ central role in family-centered palliative care is simultaneously a professional asset and a primary source of occupational risk, and Brooten et al. [28], in a longitudinal study of 63 bereaved parents, found that families’ most valued nursing behaviors—compassionate communication and emotional competence—are precisely the behaviors associated with the highest emotional cost to nurses, particularly when parental grief generates moral distress unsupported by institutional structures. Taken together, these two strands suggest that family-centered practice cannot be improved through communication training alone without also addressing the structural conditions—debriefing, institutional support—that determine whether this relational labor remains sustainable for nursing staff.

### 2.5. Structural Job Resource Deficits and Organizational Interventions for Well-Being Promotion

Beyond individual educational pathways, the psychological well-being of pediatric EOL nurses depends on structural institutional conditions that function as organizational job resources [3,4]. Three structural deficits emerge consistently from the literature: the absence of formal psychological debriefing, the lack of pediatric hospice and home-based palliative care infrastructure, and the invisibility of peer support as an organizational resource. Psychological debriefing after patient death has been shown to reduce emotional exhaustion and improve team cohesion in healthcare settings [29], yet its near-universal absence is well documented [30]. Gillman et al. [31], in a comprehensive systematic review, identified structured peer support, debriefing, and institutional well-being programs as the most effective strategies for promoting coping and resilience in oncology and palliative care nurses. Moral distress—the state of knowing the right course of action but being institutionally prevented from taking it [32],—is increasingly recognized as a distinct pathway from occupational stress to workforce attrition that educational interventions alone cannot address without accompanying organizational reform [33].

The magnitude of these structural deficits, however, is not uniform across healthcare systems. Countries with well-funded pediatric hospice networks, mandatory EOL curricula, and institutionalized debriefing protocols report comparatively lower levels of unaddressed psychosocial risk, indicating that the imbalance between job demands and job resources documented here is shaped by national health-system investment and policy choices rather than being intrinsic to pediatric oncology nursing as such. Cultural norms surrounding death, grief, and disclosure to children and families also vary across contexts and may further modulate how acutely these structural deficits are experienced by nursing staff. This variability underscores that the findings reviewed above, drawn predominantly from Anglo-Saxon, Northern European, and selected Asian healthcare systems, cannot be assumed to generalize uniformly to other national contexts, including Italy.

### 2.6. Research Gap and Rationale for the Present Study

To the best of the authors’ knowledge, no study has yet examined these phenomena through a qualitative design supported by computer-assisted quantitative textual analysis in the Italian healthcare context. The Italian context presents distinctive structural conditions—including the virtual absence of a national pediatric hospice network, a fragmented regulatory framework for continuing EOL education, and a historically underdeveloped tradition of institutional psychological support for nursing staff—that cannot be assumed to map onto findings from Anglo-Saxon or Northern European healthcare systems. The present study addresses this gap directly by combining thematic content analysis with T-Lab co-occurrence mapping on a corpus of structured interviews, generating both the depth of lived-experience documentation and a complementary lexical perspective on thematic patterns necessary to move from descriptive evidence to actionable educational and policy recommendations. A second gap concerns the application of psychological well-being frameworks to this population: existing studies have documented the psychosocial risk outcomes without systematically integrating positive well-being constructs or framing findings within a dual-process model that attends to both psychosocial risk and well-being resources. This study addresses both gaps simultaneously.

Accordingly, this study addresses the following research questions: RQ1: What organizational and educational conditions generate psychosocial risk among Italian pediatric oncology nurses? RQ2: What job resources—training, debriefing, peer support—are perceived by nurses as absent or deficient? RQ3: What does T-Lab co-occurrence analysis reveal about the semantic structure of nurses’ representations of risk and support, beyond what thematic coding alone captures.

## 3. Materials and Methods

### 3.1. Ethics Statement and Study Design

The study was conducted in accordance with the Declaration of Helsinki. Participation was voluntary; all participants signed written informed consent for audio recording and data processing. Anonymity was guaranteed through participant identification codes. Ethical approval was obtained from the Medical Direction of the Hospital Facility (11 September 2023, protocol number 0077012/23).

This is a qualitative study supported by a complementary, computer-assisted lexical analysis (T-Lab) performed on the same interview corpus [34,35], rather than a mixed-methods design in the strict sense—that is, one in which qualitative and quantitative strands are collected independently and formally integrated—or a multi-methods design, in which multiple methods are used in parallel without integration. Qualitative methodology was selected because it is particularly suited to exploring the subjective experience of occupational stress and CF in healthcare professionals, given its focus on lived experience as the primary unit of analysis [36]. The lexical analysis complements the thematic findings by mapping co-occurrence patterns among key concepts, without constituting independent validation, since both strands draw on the same corpus; the two are integrated only at the interpretation stage (see Section 5). The interview protocol was also designed to capture dimensions of relational meaning and professional satisfaction alongside risk indicators, allowing the dataset to be read through both a deficit (psychosocial risk) and a resource (psychological well-being) lens. In line with reflexive practice in qualitative research, the interviews were coded and analyzed by two work and organizational psychologists without direct clinical responsibilities on the ward, whose allowing an analytic perspective on nurses’ accounts but required active bracketing of assumptions about day-to-day nursing practice during coding. A physician and a nurse among the co-authors, both familiar with the clinical context, reviewed the resulting themes for clinical plausibility once coding was complete, without taking part in the coding process itself, so as to keep the analytic and the clinical perspective distinct. None of the researchers involved in the analysis had a prior personal or professional relationship with the participants. The two coders met after independently coding each transcript to discuss and reconcile interpretive differences, documenting key analytic decisions in an audit trail to enhance transparency and reduce the influence of individual bias on theme development.

### 3.2. Participants and Procedures

Purposive sampling was applied. Inclusion criteria were employment in the pediatric onco-hematology unit and hospice ward of the Pescara ‘Santo Spirito’ Civil Hospital, with direct involvement in caring for children with terminal onco-hematological disease; no additional exclusion criteria were applied, since all other nursing staff fell outside the population of interest by definition. Of 18 nurses approached, 15 agreed to participate and were included in the analysis; 1 declined and 2 further interviews were conducted as a pilot to test the interview guide and was not included in the final analysis. Data collection continued until thematic saturation was reached, i.e., until the coders judged, through ongoing comparison across interviews, that no substantially new codes or themes were emerging; this judgment was reached by consensus between the two coders after the final interviews. The study period was September–October 2023. Participants included pediatric specialists and nurses transitioning from surgery, adult hematology, and general medicine, reflecting diverse prior EOL exposure. Data were collected through structured individual face-to-face interviews comprising a sociodemographic section and a structured guide with five main question areas: (1) quality of nursing care; (2) achievement of maximum health; (3) relationship with parents; (4) coping with the child’s death; and (5) impact on personal and professional life (see Appendix A for the full interview guide). The guide was pilot-tested on two nurses not included in the final sample. All interviews were audio-recorded and fully transcribed verbatim. The two coders performed conventional (inductive) qualitative content analysis [36]. An initial codebook was developed inductively from the first five transcripts and then applied to the remaining ten; coding discrepancies, affecting fewer than 10% of codes, were resolved through consensus discussion between the two coders, with a third researcher acting as arbiter when needed. Themes were finalized through iterative comparison across all fifteen transcripts (stepwise replication); resulting themes were subsequently reviewed for clinical plausibility by a physician and a nurse among the co-authors, as detailed in Section 3.1. Quantitative textual analysis was performed with T-Lab (2024) [37]. Transcripts were pre-processed by removing filler words and standardizing spelling variants; T-Lab’s automatic lemmatization was applied and manually verified by the research team; interviewer utterances and backchannel tokens were excluded via a custom stop-word list; lemmas occurring fewer than five times in the corpus were excluded from co-occurrence analysis. Co-occurrence was computed at the elementary-context-unit level (sentence-level window) using the ‘Word Association’ function, quantifying the strength of association between key lemmas with the Cosine coefficient and testing its statistical significance via chi-square (χ^2^); given the exploratory nature of this analysis, associations are reported at *p* < 0.05 uncorrected for multiple comparisons, consistent with standard T-Lab reporting conventions [35].

## 4. Results

### 4.1. Descriptives

Fifteen nurses, 3 men and 12 women, participated (Table 1). Professional profiles were heterogeneous: seniority ranged from approximately 2 to over 30 years, and several participants had previously worked in adult hematology, surgery, or hospice settings. This diversity in prior EOL exposure is itself a structural indicator of the absence of standardized preparatory training, consistent with the educational deficits documented in Section 2.3.

### 4.2. Summary of Themes

Seven themes emerged from the analysis (Table 2). CF and occupational stress pervaded Themes 1, 6, and 7 as primary constructs, and appeared as secondary dimensions in Themes 2, 3, and 5. Themes 3 (child management) and 4 (maximum achievable health) additionally surfaced as sources of relational meaning and professional satisfaction, representing the positive well-being counterpart to the risk-dominant pattern—a dimension discussed in Section 4.3 below.

### 4.3. Thematic Findings

#### 4.3.1. Quality of Nursing Care

Quality of nursing care was rated as ‘good’ by five participants, ‘excellent’ by four, and ‘sufficient’ by two. Nearly all identified a structural training gap as the principal factor limiting care quality and, simultaneously, as an independent source of occupational stress. P6 articulated the core problem: “Anyone could care procedurally for a terminal patient after a basic degree. The difficulty lies in emotional care, in communication, in recognizing one need over another—for that you need preparation.” Most acquired EOL competencies informally: “I had twenty years of surgery behind me, but in hospice I had to start from zero” (P1). The protective effect of formal education are consistent with P13, whose Master’s training directly reduced distress and strengthened professional identity, replicating Jeong et al. [23] at the program level.

#### 4.3.2. Child’s Needs

Pain management was the most frequently cited child need (P2, P4, P9, P12, P14, P15). A recurring occupational stressor was parental resistance to palliative sedation: “When you start sedation, you know there is no turning back” (P8). Structural deficits—absence of dedicated pediatric hospice, inadequate home care—functioned as stress amplifiers, compelling nurses to manage terminal care without appropriate infrastructure [15].

#### 4.3.3. Child Management

All participants described a strategy of deliberate ‘presence-absence’ in the terminal phase: entering rooms gently, conveying support through gestures rather than words. P6 termed this “a passive support, a presence-absence.” P12 reported: “I learned in these 12 years that silence is the best strategy.” These relational adaptations, acquired exclusively through individual experience, are consistent with emotional labor theory [38]. Notably, these same strategies also constituted a source of professional meaning for several nurses: the capacity to ‘be present’ at the moment of death was described by P4 and P13 as a marker of professional identity and a form of compassion satisfaction. This dual function—occupational risk and source of meaning—is characteristic of the high-demand, high-significance nature of EOL nursing [18,22].

#### 4.3.4. Maximum Achievable Health

The principle of ‘quality over quantity of life’ was universally endorsed. The dominant occupational stressor was time scarcity: “I want to have more time for them” emerged across six participants (P2, P3, P4, P5, P9, P15). This inability generates moral distress [32]—a recognized pathway toward CF and burnout [33]. Conversely, nurses who described achieving quality-focused care outcomes reported an associated sense of purpose and satisfaction; read post hoc through Ryff’s [16] model, this pattern is consistent with the ‘purpose in life’ dimension of psychological well-being, though the interview guide was not designed a priori to assess this construct. P1’s statement “We work on quality, not quantity” encapsulates a value orientation that, when institutionally supported, functions as a protective psychological resource.

#### 4.3.5. Parental Relationship

Relationships with parents were described as the most challenging relational dimension. Trust required active construction over time (P13). Nursing-led bereavement follow-up was absent for 10 of 15 participants. The absence of structured follow-up generates ‘role helplessness’—knowing what should be done but being institutionally unable to do it [3]—a direct occupational stressor consistent with Macnab et al. [39] and Macdonald et al. [40].

#### 4.3.6. Coping with a Child’s Death

The death of a child was described as non-normalizable by all participants: “The death of a child is unacceptable” (P1). The near-universal absence of formal psychological debriefing was identified as the central institutional failure. P8, who left the unit after 12 years, stated: “I thought I had overcome it—but speaking about it today, I realize I have not.” P7 articulated the progressive depletion model: “We consume ourselves little by little”—consistent with cumulative empathic erosion frameworks.

#### 4.3.7. Impact on Personal and Professional Life

Most nurses reported emotional intrusion—involuntary recollection of deceased patients during personal time (P2, P6, P11)—and emotional contagion affecting family relationships (P3, P8, P10). P3, with over 30 years of experience, provided the most clinically significant description: “You hold—until perhaps one day you cannot hold anymore. You are like a sponge that absorbs; one day the sponge is no longer usable.” In contrast, P15 described an adaptive trajectory characterized by active peer support, deliberate boundary-setting, and a sustained sense of professional meaning—illustrating that psychological well-being is achievable in this setting when organizational and relational resources are available. This case functions as a within-sample demonstration of the positive pole of the well-being continuum, consistent with compassion satisfaction frameworks [17,18].

### 4.4. T-Lab Quantitative Analysis

T-Lab quantitative textual analysis of the full interview corpus provided complementary lexical evidence supporting and extending the qualitative findings, offering a frequency-based mapping of the semantic structure underlying nurses’ representations of pediatric EOL care. The two most frequent lemmas were child (n = 235) and parent (n = 110). This result, while not unexpected, carries an unambiguous theoretical implication: it is impossible to conceive of care for the dying child independently of care for the parent and the broader family system. The following subsections present detailed co-occurrence results for each of the six lemmas interrogated: child, parent, care, need, death, and support.

**Figure 1:**
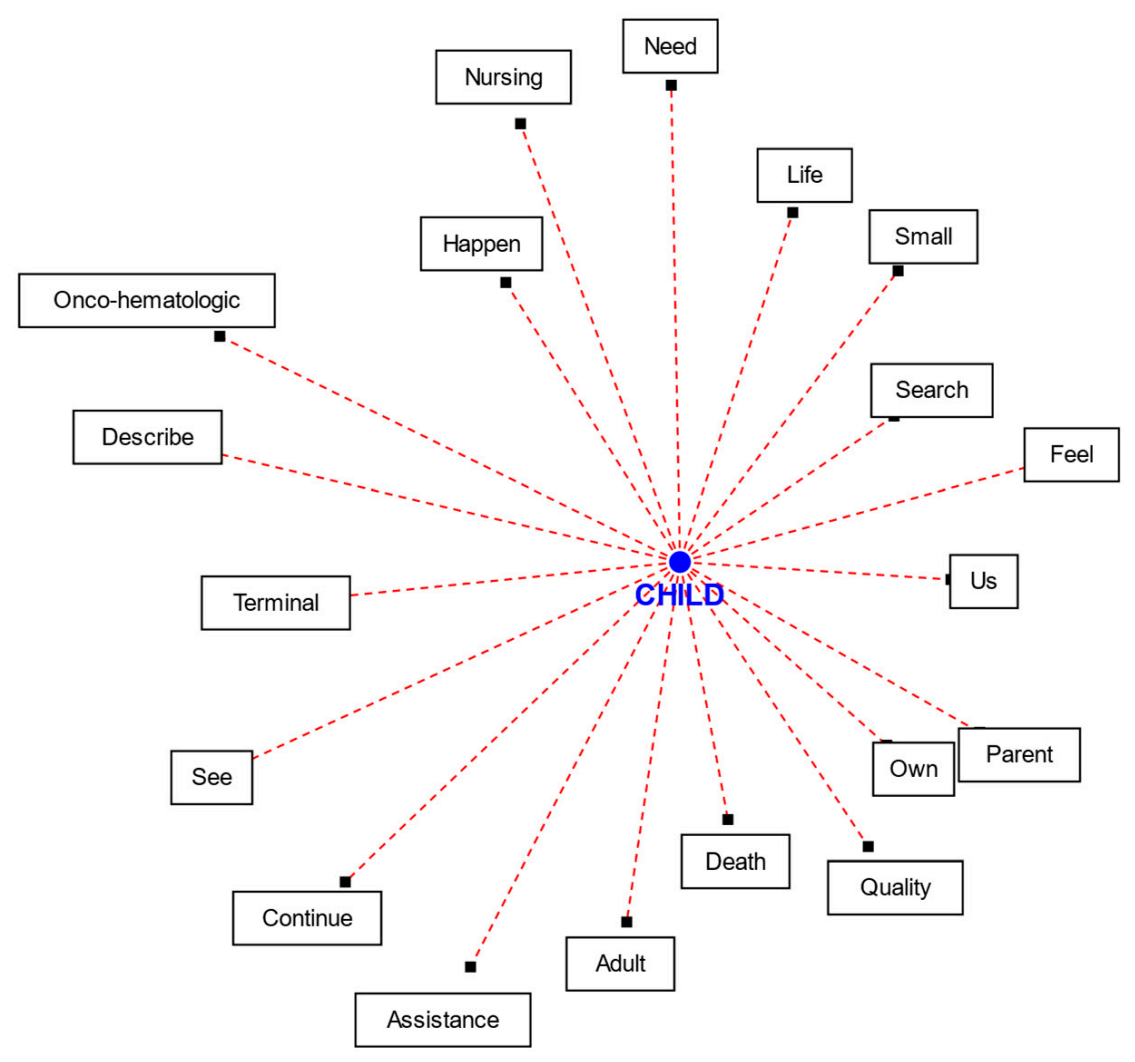
Co-occurrence with lemma “Child”.

Child (n = 235, Figure 1). As shown in Table 3, the strongest association is with parent (Cosine = 0.442; χ^2^ = 12.15; *p* < 0.001), indicating the structural inseparability of the child–parent dyad as the relational core of EOL nursing practice and the primary generator of occupational risk.

The second-strongest association is with death (Cosine = 0.419; *p* < 0.001), indicating that in nurses’ mental representation the child is cognitively inseparable from death—a co-occurrence that maps directly onto the progressive depletion model [4,11] and explains why this specialty generates compassion fatigue at rates higher than other nursing contexts. The association with terminal (Cosine = 0.326; *p* < 0.001) supports that discourse about the child is predominantly organized around the terminal phase [19,21]. Particularly significant for occupational sustainability are the associations with coping (Cosine = 0.245; *p* < 0.001), work-related (Cosine = 0.223; *p* = 0.029), and follow-up (Cosine = 0.192; *p* = 0.030): when nurses speak of the child, the absence of structured coping tools and post-bereavement pathways surfaces as a semantically proximate concern, a direct quantitative marker of role helplessness and a primary target for educational intervention [23].

**Figure 2:**
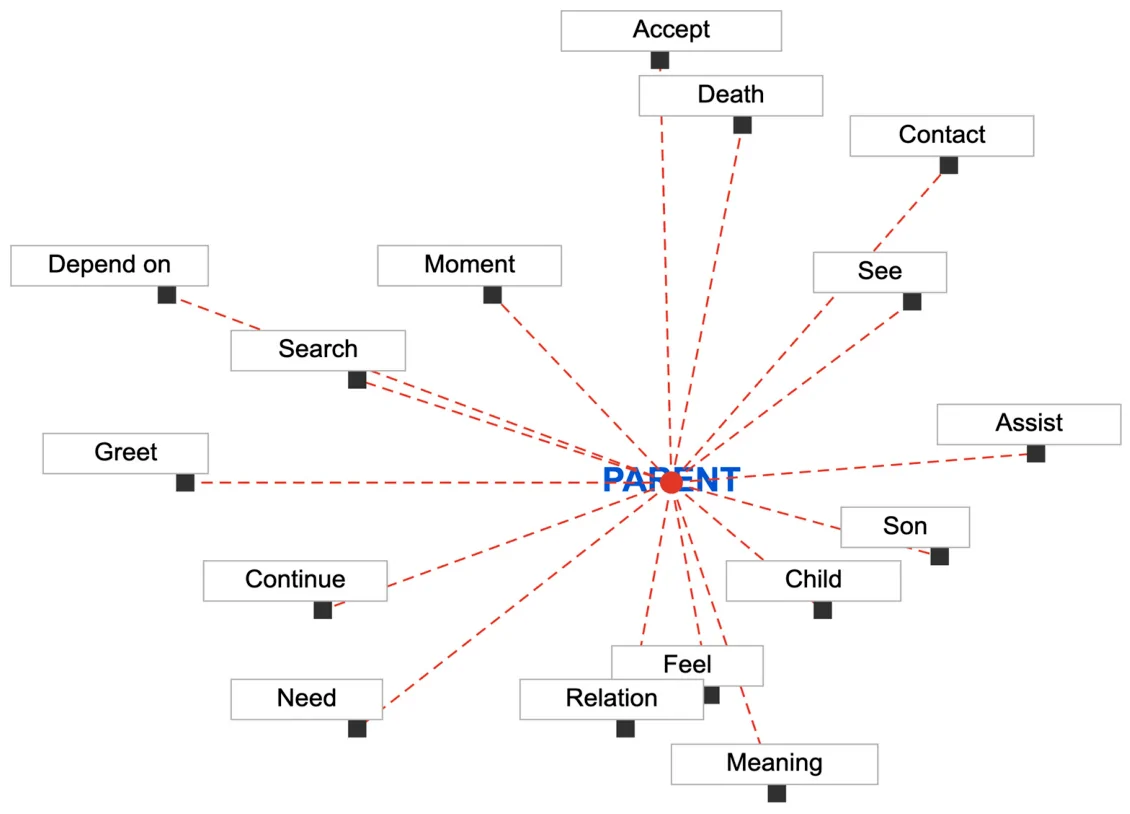
Co-occurrences with Lemma “Parents”.

Parent (n = 110, Figure 2. Table 4).

The mutual primacy of the child–parent association (Cosine = 0.442; *p* < 0.001) supports the bidirectionality of this relational bond: the dyad functions as a single, indivisible unit of care [26,27]. The associations with relate (Cosine = 0.357; *p* < 0.001) and feel (Cosine = 0.302; *p* < 0.001) indicate that the parent–nurse relationship is primarily conceptualized in affective and relational terms, consistent with emotional labor theory [38]. The association with moment (Cosine = 0.280; *p* = 0.001) and continue (Cosine = 0.301; *p* < 0.001) suggests nurses’ representations are anchored to specific temporal moments of loss and to the question of continuity of care beyond them. The association with death (Cosine = 0.277; *p* = 0.012) supports the interpretation that bereavement is a defining dimension of the parent–nurse relationship. The association with need (Cosine = 0.193) did not reach statistical significance (χ^2^ = 0.52, *p* = 0.471; Table 4) and is therefore not interpreted as a reliable pattern; it is reported here only for completeness.

**Figure 3:**
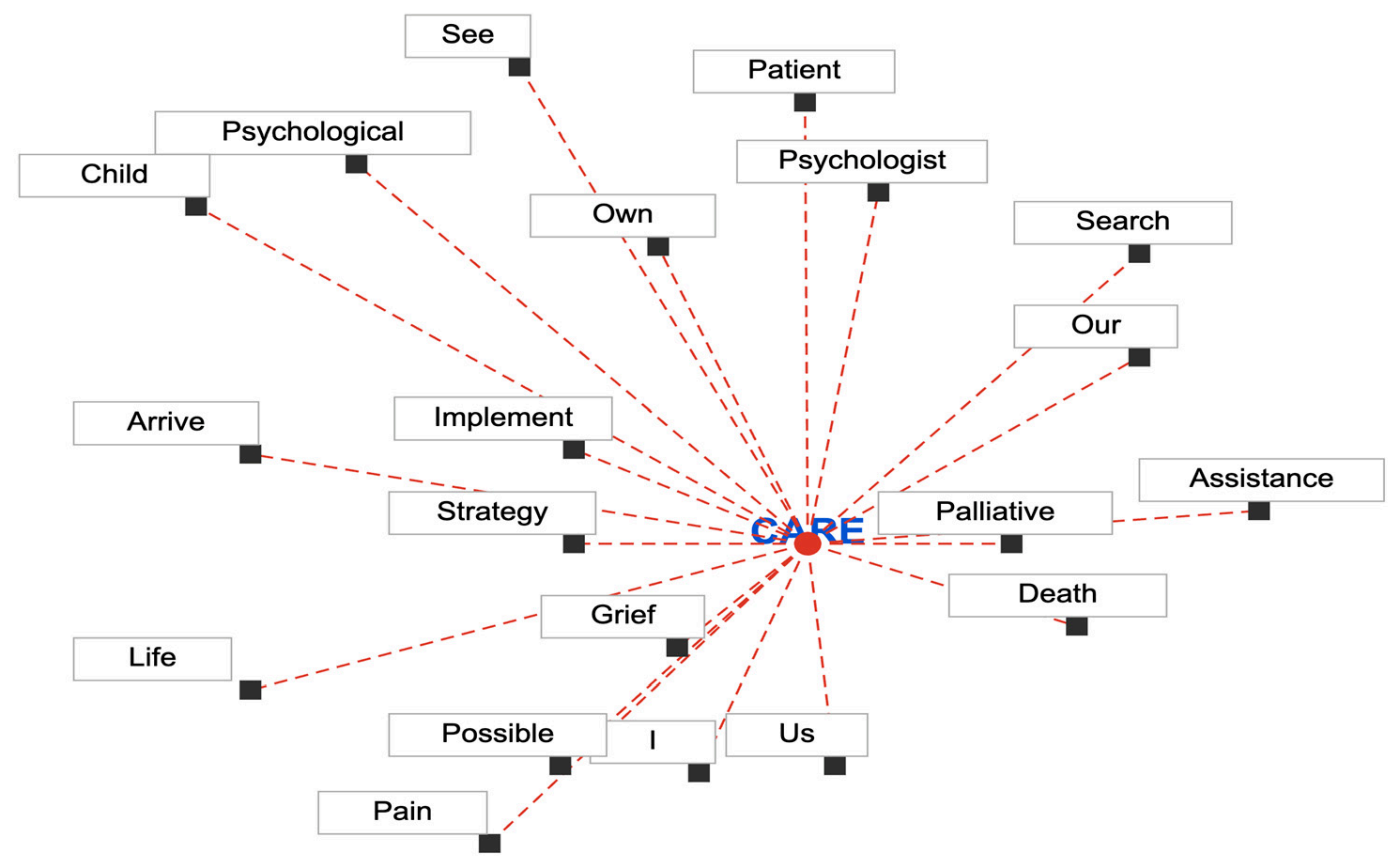
Co-occurrences with the lemma “care”.

Care (n = 52). Figure 3.

The strongest association is with palliative (Cosine = 0.514; *p* < 0.001) (Table 5). This indicates that in nurses’ lexical representation the concept of care is almost exclusively associated with its palliative dimension, indicating that nurses in this specialty have internalized a care philosophy oriented toward comfort and dignity rather than cure [1]. The strong associations with grief (Cosine = 0.438; *p* < 0.001), strategy (Cosine = 0.407; *p* < 0.001), and implement (Cosine = 0.406; *p* < 0.001) reveal that care is inseparably linked to grief management and the active implementation of coping strategies. The association with psychologist (Cosine = 0.191; *p* = 0.004) supports the qualitative finding: psychological support in post-bereavement care is recognized as essential but exclusively conceptualized as a specialist clinical function, reinforcing the conceptual gap identified in the analysis of the lemma support.

**Figure 4:**
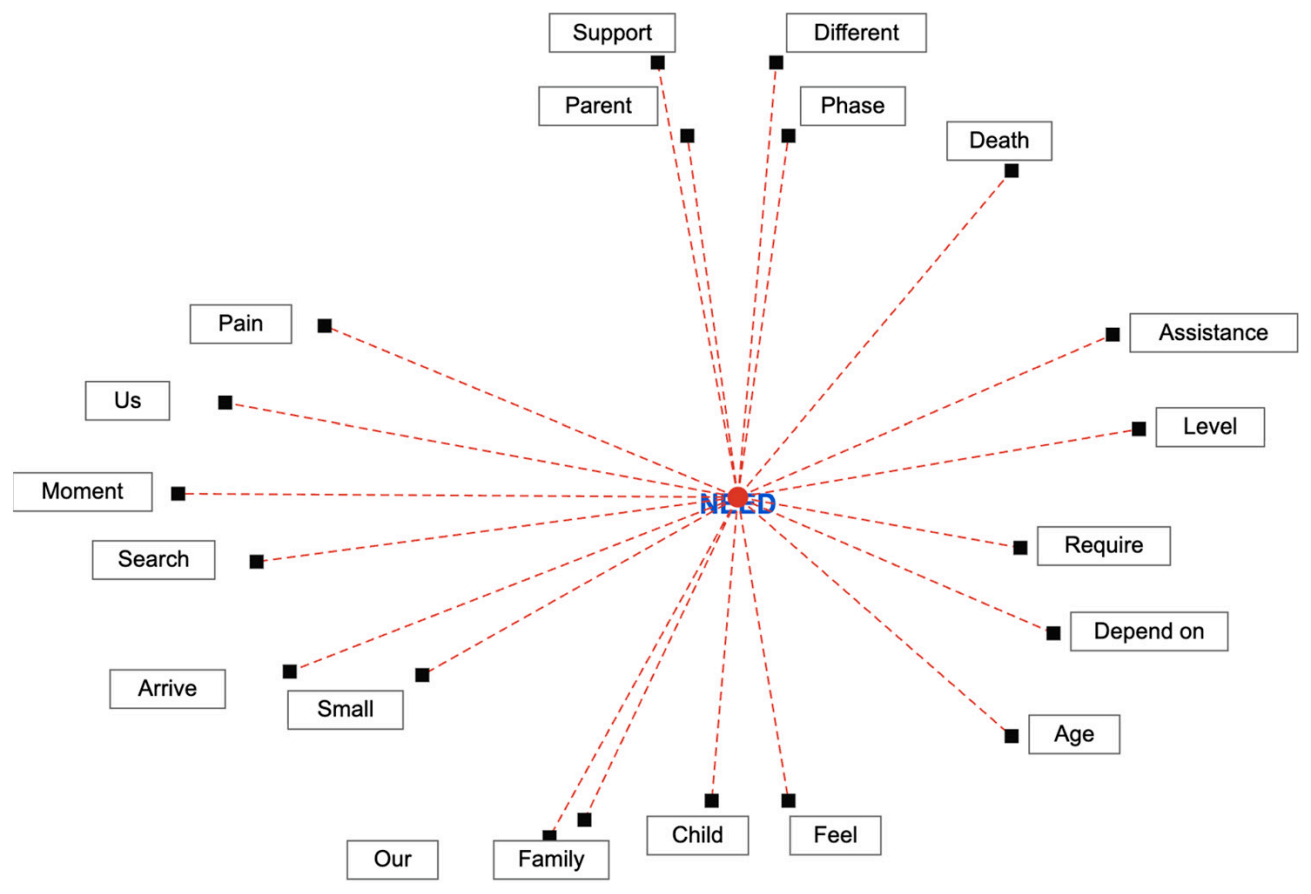
Co-occurrences with the lemma “need”.

Need (n = 55, Figure 4.)

Need is most frequently associated with child (Cosine = 0.264), though this association did not reach statistical significance (χ^2^ = 0.51, *p* = 0.473) and should be read as an exploratory pattern rather than a confirmed finding. The associations with parent (Cosine = 0.193) and family (Cosine = 0.179) likewise did not reach significance; while suggestive of a broader family-centered orientation [27], these patterns require cautious interpretation given the lack of statistical support. The association with pain (Cosine = 0.197; *p* = 0.017) is consistent with the centrality of symptom management as the most immediate and recognizable expression of the child’s need, consistent with the qualitative finding that pain control is the most frequently cited care priority. The associations with phase (Cosine = 0.191; *p* = 0.004) and depend (Cosine = 0.186; *p* = 0.006) indicate that nurses recognize the phase-contingent and context-dependent nature of needs—consistent with the adaptive, individualized care documented in thematic analysis and with age-specific communication strategies described by Ruhe et al. [24]. All the associations can be found in Table 6.

**Figure 5:**
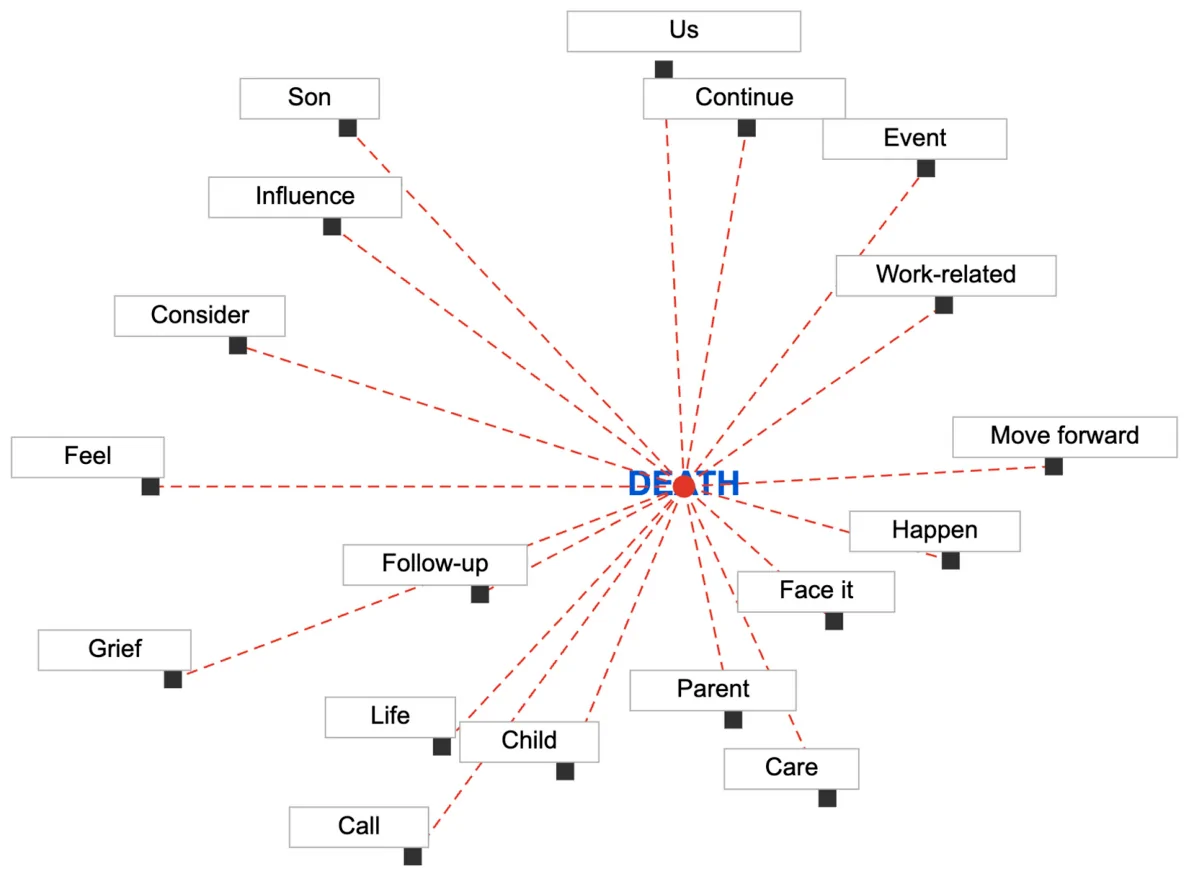
Co-occurrences with the lemma “death”.

Death (n = 68, Figure 5)

The strongest association is with face it (coping with) (Cosine = 0.455; *p* < 0.001)—the highest coefficient recorded for this lemma and among the highest in the entire corpus (Table 7). This is not an association of mastery or resolution: it is an association of chronic, unresolved tension. Combined with the near-universal absence of structured debriefing documented, this pattern constitutes the most direct quantitative indicator of compassion fatigue as a structural occupational outcome [7,8]. The association with follow-up (Cosine = 0.389; *p* < 0.001) reveals that nurses cognitively link the death of a child to the question of post-bereavement follow-up and, implicitly, to its absence: this co-occurrence functions as a quantitative marker of role helplessness and a primary target for educational intervention [23]. The associations with work-related (Cosine = 0.336; *p* < 0.001) and continue (Cosine = 0.353; *p* < 0.001) are consistent with that of child death has direct and lasting effects on nurses’ professional functioning—consistent with the progressive depletion model described by P3 and P7 in Section 4.3.7 and with El-Labban et al.’s [9] quantitative evidence linking client-related burnout to psychological distress. Table 7 shows the most important association with the word Death.

**Figure 6:**
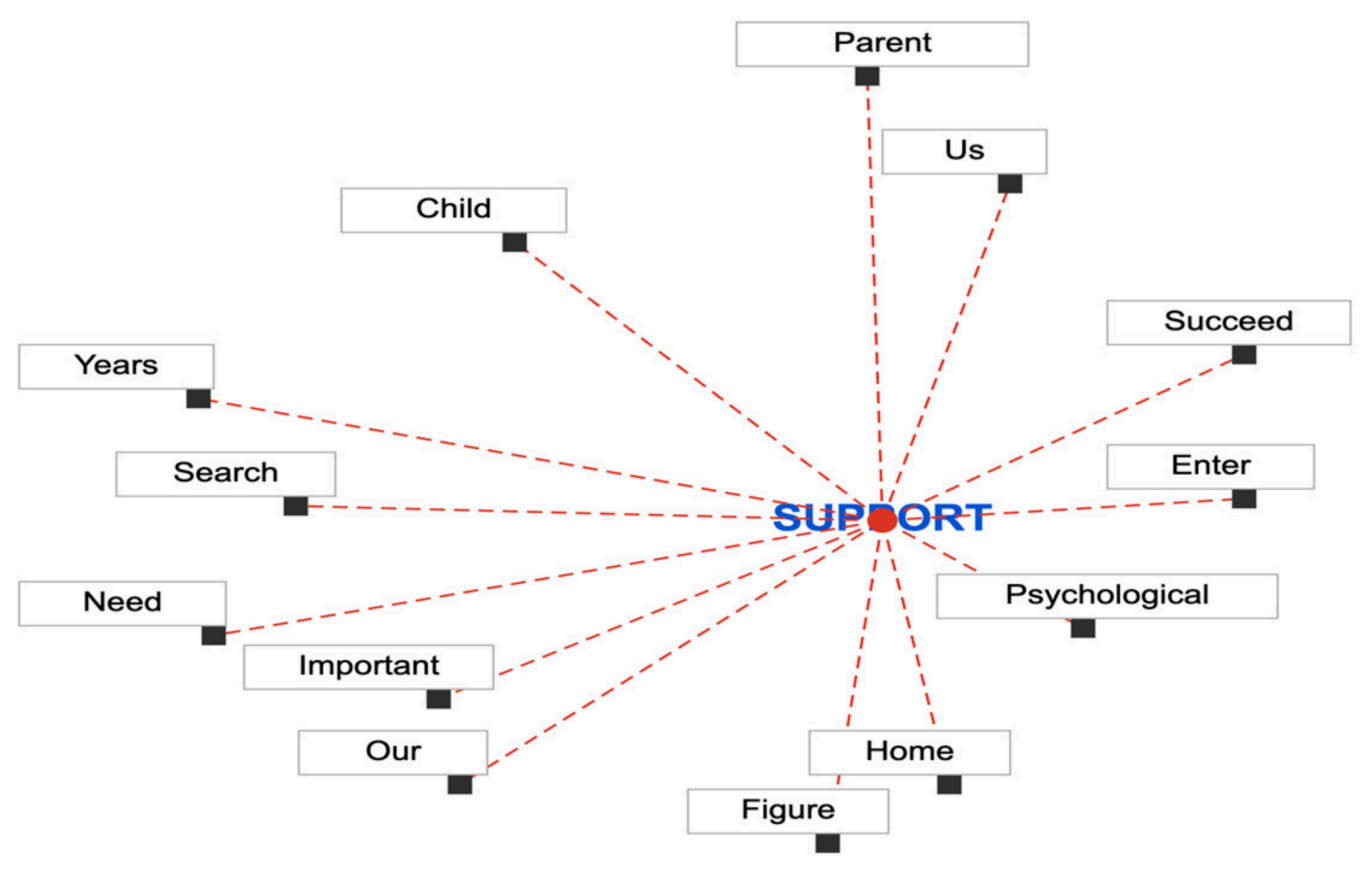
Co-occurrences with the word “support”.

Support (n = 24). Figure 6.

Despite its relatively low frequency, this lemma carries the highest diagnostic significance for occupational sustainability policy.

Its dominant association is with psychological (Cosine = 0.379; *p* < 0.001) (Table 8), indicating that support is almost exclusively conceptualized in its clinical-individual dimension—as a specialist psychological service—with no semantic proximity to peer-level, team-level, or organizational support structures. This is the critical conceptual gap identified in this study: nurses recognize the need for support but lack the vocabulary and organizational framework to conceptualize it beyond the dyadic psychologist–patient relationship. The association with figure (Cosine = 0.225; *p* < 0.001) reinforces this finding: support is conceptualized through the lens of a professional figure rather than a systemic or structural mechanism. The absence of any association between support and terms such as colleague, team, or peer in the co-occurrence network is itself informative: it supports that informal peer sharing—described as the most used coping mechanism in the qualitative analysis—has not been integrated into nurses’ formal professional vocabulary. This invisibility represents both the most significant organizational gap identified in this study and the most immediately actionable target for educational and institutional reform.

Taken together, the T-Lab analysis provides complementary lexical support for all seven qualitative themes and surfaces three overarching structural patterns with direct implications for psychosocial risk and psychological well-being. First, the child–parent dyad is the relational core of EOL nursing practice, generating both professional meaning and compassion fatigue risk. Second, death activates a cluster of unresolved occupational needs—coping without tools, follow-up without pathways, grief without institutional processing—that collectively constitute the structural mechanism through which compassion fatigue is produced and sustained. Third, support is exclusively conceptualized in individual-clinical terms, rendering peer-level and team-level structures semantically and organizationally invisible. These three patterns define both the problem—psychosocial risk rooted in relational intensity without institutional job resources—and its educational solution: structured curricula, formalized peer support, and systemic bereavement protocols.

This study documents, through a qualitative descriptive design supported by complementary textual analysis, that pediatric oncology nurses in Italy operate under conditions of significant psychosocial risk and compromised psychological well-being—and that the organizational, educational, and infrastructural conditions required to address, prevent, and mitigate these conditions remain structurally absent from the Italian healthcare context.”

## 5. Discussion

This study contributes to the occupational health and psychosocial risk literature by documenting, through a qualitative design supported by complementary lexical analysis, the structural conditions that generate psychosocial risk and compromise psychological well-being among pediatric oncology nurses in the Italian healthcare context. The findings support the interpretation that compassion fatigue and occupational stress in this specialty are not individual clinical vulnerabilities but systemic outcomes of a workforce operating under a chronic structural imbalance between high job demands and deficient job resources. Beyond this substantive contribution, the study also illustrates, as a secondary methodological aim, how thematic and lexical co-occurrence analyses can be jointly used to assess psychosocial risk in high-intensity care settings.

The Job Demands–Resources (JD–R) model [3,4] provides a theoretically integrative framework for interpreting the study’s findings. Within this model, the chronic mismatch documented here—high relational and emotional job demands (child death, parental grief containment, moral distress) combined with minimal job resources (absent debriefing, no peer support structures, inadequate hospice infrastructure)—constitutes the structural precondition for both burnout and the erosion of psychological well-being. The T-Lab finding that ‘support’ co-occurs exclusively with ‘psychological’ and never with ‘team’ or ‘peer’ is directly interpretable within JD–R as evidence of a job resources deficit at the organizational level: nurses recognize the need for support but can only conceptualize it as an individual-clinical intervention, not as a systemic organizational resource. This finding has direct practical implications: building job resources—formalized peer support, debriefing protocols, recognition of relational labor—is not an ‘add-on’ to clinical care but the primary lever for promoting and sustaining psychological well-being in this workforce.

CF emerged not as an episodic or individual vulnerability but as a structural psychosocial risk outcome [18]. The progressive depletion model described by P7 and P3 maps directly onto theoretical frameworks of CF as cumulative empathic erosion [11,41] and is consistent with the grounded theory findings of Gurcan et al. [42] and the qualitative findings of Bian et al. [43]. The T-Lab evidence that ‘death’ co-occurs strongly with ‘coping’ (Cosine 0.45) indicates that patient death functions as both an acute stressor and a chronic occupational risk factor.

A distinctive contribution of this study is its documentation of psychosocial risk within an ‘evolving workplace’ context. The Italian pediatric palliative care system is undergoing structural transformation, with national policy [5] promoting a shift toward home-based and hospice models that have not yet been accompanied by commensurate investment in nursing workforce preparation, role definition, or psychological support infrastructure. The nurses in this study operated at the frontier of this transition—managing terminal care in settings ill-equipped for its demands, without the organizational job resources that psychological well-being in EOL care requires. This ‘evolving workplace’ dimension amplifies pre-existing psychosocial risks and constitutes an additional target for policy intervention that extends beyond individual CF prevention to systemic investment in job resources and well-being infrastructure.

The protective effect of structured education was demonstrated empirically within the sample: P13 and P4 reported significantly lower occupational stress and stronger professional identity than peers without postgraduate palliative training, consistent with prior evidence on the role of structured introduction programs in shaping novice oncology nurses’ preparedness for EOL care [44]. According to recent literature [45], the absence of mandatory, structured EOL curricula in Italian nursing education constitutes a primary job resource deficit at the educational level it deprives nurses of the competence and emotional preparedness that JD–R research identifies as foundational buffers against CF accumulation and psychological distress [3,4].

The findings of this study support a shift from a purely deficit-reduction model of occupational health intervention toward a well-being promotion orientation. The within-sample evidence that P15 achieved psychological adaptation through active peer support and meaning-making, and that Themes 3 and 4 generated relational satisfaction alongside risk, demonstrates that compassion satisfaction is achievable in this setting—but only when organizational conditions make it possible. Building these conditions requires not only eliminating structural risk factors (absent debriefing, inadequate training) but actively investing in the job resources that generate and sustain well-being: formalized peer support groups, structured bereavement follow-up as a recognized nursing competency, and institutional recognition of the relational dimension of EOL care as a source of professional meaning, not merely occupational burden.

The study’s limitations include its single-center design and the absence of validated psychometric instruments for CF (e.g., ProQOL) and burnout (e.g., MBI or Copenhagen Burnout Inventory. In addition, Ryff’s model and the other auxiliary constructs discussed in Section 2 (compassion satisfaction, emotional labor, moral distress) were applied as post hoc interpretive lenses rather than built a priori into the interview guide or coding scheme, and findings interpreted through them should be read as exploratory rather than confirmatory. Because compassion fatigue, psychological well-being, and reported intention to leave were not assessed with validated psychometric or clinical instruments, related findings should be interpreted as reflecting participants’ subjective perceptions rather than clinically or psychometrically confirmed states. Moreover, since the thematic and T-Lab analyses were performed on the same interview corpus, they do not constitute independent validation of one another; T-Lab co-occurrence patterns may also have been shaped by the structure and wording of the interview guide itself, and the lexical analysis remains exploratory. Associations reported in the T-Lab analysis were tested at *p* < 0.05 uncorrected for multiple comparisons, consistent with standard T-Lab conventions but a further limitation to bear in mind when interpreting individual associations.

Future research should integrate the ProQOL’s Compassion Satisfaction subscale to capture the positive well-being dimension alongside CF and Secondary Traumatic Stress and should apply the JD–R model as an explicit analytical framework to enable systematic comparison of demands and resources across settings and nursing specialties. Multi-center samples and quasi-experimental intervention designs are also recommended. Studies examining the evolving workplace conditions specific to transitional healthcare systems—including the Italian context of expanding home-based and hospice palliative care—would further advance understanding of how organizational transformation interacts with psychosocial risk and well-being outcomes in high-intensity nursing specialties.

## 6. Conclusions

This study suggests that the occupational unsustainability observed at the institution studied may be structurally generated and, in principle, addressable through organizational change. Compassion fatigue-related experiences, moral distress, and reported intention to leave in this specialty appear to be associated with organizational and educational conditions rather than solely reflecting individual clinical vulnerabilities [31,46]. These findings are specific to the single institution studied and should not be generalized to the national level without further multi-center research; at a broader level, they may point to organizational and educational conditions worth further investigation, with possible relevance to SDG 3 (Good Health and Well-Being) and SDG 8 (Decent Work and Economic Growth).

At the individual and educational level, the integration of CF prevention, grief education, moral distress management, and EOL communication modules into both undergraduate curricula and continuing professional development programs is urgently required. At the organizational level, psychological debriefing, peer-support groups and systematic well-being programmes must be institutionalized as organizational standards, not delegated to informal peer sharing [29,30,31]. At the systemic and policy level, investment in pediatric hospice and home-based palliative care infrastructure is a prerequisite for enabling sustainable EOL nursing roles in practice. These recommendations are framed not only in terms of risk reduction but of active psychological well-being promotion: building the job resources—peer support, institutional recognition, structured grief processing—that enable nurses to experience compassion satisfaction alongside the inherent demands of EOL care, and to sustain meaningful professional engagement in an evolving healthcare landscape, reframing the relational and embodied dimension of nursing care as a source of professional meaning rather than mere occupational burden [47].

These recommendations are consistent with the WHO Global Strategy on Human Resources for Health: Workforce 2030 [6]. Based on the findings of this single-center study, the occupational sustainability of pediatric oncology nursing may be usefully framed as a patient safety concern, a health system resilience factor, and health coverage for the most vulnerable populations.

## Figures and Tables

**Table 1:** Participant profiles with occupational stress and compassion fatigue indicators (N = 15).

ID	Gender	Setting	Seniority	CF/Occupational Stress Indicator
P1	M	Hospice	20 yr	Explicit burnout risk; departure threshold described
P2	F	Hospice	4 yr	Avoids post-bereavement contact; emotional equilibrium at risk
P3	F	Hospice/Onc.	>30 yr	30+ yr accumulated grief; ongoing unresolved emotional weight
P4	M	Hospice/Onc.	<10 yr	Master’s in palliative care as response to occupational stress
P5	F	Ped. Oncol.	5 yr	SICP access barriers; structural inadequacy as stress amplifier
P6	F	Ped. Oncol.	10 yr	Sought external psychoanalytic support; CF trajectory explicit
P7	M	Ped. Oncol.	16 yr	‘We consume ourselves little by little’; no institutional support
P8	F	Ped. Oncol.	23 yr	Left unit after 12 yr; unresolved grief persists post-departure
P9	F	Ped. Oncol.	25 yr	Structural isolation; absent hospice/home care as stressors
P10	F	Ped. Oncol.	20 yr	Emotional intrusion into private life; depressive episodes
P11	F	Ped. Oncol.	12 yr	Internalizes child death as professional failure; identity threat
P12	F	Ped. Oncol.	30 yr	Protective silence acquired through experience only
P13	F	Ped. Oncol.	10 yr	Master’s training linked to stress reduction and professional identity
P14	F	Ped. Oncol.	2 yr	‘No pain’ analgesic culture as protective factor; reduced moral distress
P15	F	Ped. Onc./Hem.	13 yr	Optimal adaptation; active peer support as coping resource

CF = compassion fatigue; Ped. Oncol. = Pediatric Oncology; Hem. = Hematology.

**Table 2:** Summary of emergent themes with compassion fatigue and occupational stress relevance (N = 15).

Theme	Key Sub-Themes	N	CF/Stress Relevance	Representative Quote
1. Quality of nursing care	Training gap; procedural vs. emotional care	15/15	High	“For emotional care you need preparation” (P6)
2. Child’s needs	Pain; FCC; play; home care; psych. support	15/15	Moderate-High	“The child must never feel pain” (P2, 4, 9, 12, 14, 15)
3. Child management	Trust; silence; nonverbal comms.; empathy	15/15	Moderate	“Silence is the best strategy” (P12)
4. Maximum achievable health	Quality vs. quantity; play; time; teamwork	15/15	Moderate	“We work on quality, not quantity” (P1)
5. Parental relationship	Trust-building; grief; absent follow-up	15/15	Moderate-High	“Follow-up is absent from nursing” (P2, 5–10, 12, 14, 15)
6. Coping with death	Emotional involvement; grief; absent debriefing	15/15	High	“We consume ourselves little by little” (P7)
7. Personal/professional impact	Burnout risk; emotional intrusion; detachment	15/15	High	“You hold—until you cannot hold anymore” (P3)

FCC = Family Centered Care; CF = compassion fatigue. Note: N indicates the number of participants whose transcript contained at least one reference to the theme; coding was non-exclusive, meaning themes could co-occur within the same transcript, which is why all seven themes show N = 15/15. Sub-theme-level frequencies, where distinguishable, are reported in the narrative discussion of each theme in Section 4.3 (e.g., the sub-theme ‘pain’ under Theme 2 was explicitly raised by 6 of 15 participants: P2, P4, P9, P12, P14, P15; the sub-theme ‘absent follow-up’ under Theme 5 was raised by 9 of 15 participants: P2, P5–P10, P12, P14, P15).

**Table 3:** Associations with the lemma “Child”.

LEMMA_B	COEFF	CE_B	CE_AB	CHI2	(*p*)
parent	0.441599	110	71	12.15354	<0.001
death	0.419264	68	53	24.82733	<0.001
terminal	0.326164	36	30	17.32719	<0.001
life	0.314814	62	38	3.641683	0.056
quality	0.284343	19	19	19.80044	<0.001
assistance	0.271385	52	30	1.383879	0.239
need	0.26388	55	30	0.514786	0.473
small	0.261384	18	17	14.78859	<0.001
feel	0.25569	44	26	1.60478	0.205
own	0.254778	59	30	0.019382	0.889
adult	0.254412	19	17	12.341	<0.001
onco-hematologic	0.25314	17	16	13.73198	<0.001
nursing	0.247885	25	19	7.139776	0.008
face it	0.244623	16	15	12.68172	<0.001
relate	0.244079	14	14	14.42982	<0.001
understand	0.240249	39	23	1.370099	0.242
succeed	0.2293	59	27	0.484556	0.486
influence	0.224481	19	15	6.636714	0.01
work-related	0.222523	22	16	4.768669	0.029
relationship	0.221901	28	18	2.430511	0.119

Note: LEMMA_B: base form of element B as it appears in the analysis. COEFF: association coefficient between elements A and B. CE_B and CE_AB: expected counts; CHI2: chi-square statistic (χ^2^); (*p*): *p*-value.

**Table 4:** Associations with the lemma “Parent”.

LEMMA_B	COEFF	CE_B	CE_AB	CHI2	(*p*)
child	0.441599	235	71	12.15354	<0.001
relate	0.356753	14	14	47.22488	<0.001
feel	0.301854	44	21	16.02051	<0.001
continue	0.300623	17	13	27.70686	<0.001
search	0.288276	92	29	4.204722	0.04
moment	0.280374	51	21	10.07947	0.001
death	0.277498	68	24	6.269529	0.012
see	0.24025	63	20	2.823979	0.093
son	0.218739	19	10	9.435253	0.002
relationship	0.216225	28	12	6.284956	0.012
need	0.192847	55	15	0.519986	0.471

Note: LEMMA_B: base form of element B as it appears in the analysis. COEFF: association coefficient between elements A and B. CE_B and CE_AB: expected counts; CHI2: chi-square statistic (χ^2^); (*p*): *p*-value.

**Table 5:** Associations with the word “Care”.

LEMMA_B	COEFF	CE_B	CE_AB	CHI2	(*p*)
palliative	0.514496	9	9	117.6649	<0.001
grief	0.438357	30	14	74.25199	<0.001
strategy	0.406745	40	15	59.67996	<0.001
implement	0.40584	35	14	60.49962	<0.001
psychologist	0.191079	29	6	8.338627	0.004
our	0.173931	35	6	5.532868	0.019

Note: LEMMA_B: base form of element B as it appears in the analysis. COEFF: association coefficient between elements A and B. CE_B and CE_AB: expected counts; CHI2: chi-square statistic (χ^2^); (*p*): *p*-value.

**Table 6:** Associations with the word “Need”.

LEMMA_B	COEFF	CE_B	CE_AB	CHI2	p
child	0.26388	235	30	0.514786	0.473
small	0.222475	18	7	13.3885	<0.001
pain	0.196865	38	9	5.744435	0.017
parent	0.192847	110	15	0.519986	0.471
phase	0.190693	18	6	8.475753	0.004
assistance	0.186989	52	10	3.207326	0.073
depend on	0.185606	19	6	7.571007	0.006
family	0.17893	46	9	3.051133	0.081

Note: LEMMA_B: base form of element B as it appears in the analysis. COEFF: association coefficient between elements A and B. CE_B and CE_AB: expected counts; CHI2: chi-square statistic (χ^2^); (*p*): *p*-value.

**Table 7:** Associations with the word “Death”.

LEMMA_B	COEFF	CE_B	CE_AB	CHI2	(*p*)
face it	0.454754	16	15	84.13389	<0.001
child	0.419264	235	53	24.82733	<0.001
follow-up	0.388922	14	12	59.18957	<0.001
continue	0.352941	17	12	44.88973	<0.001
work-related	0.336107	22	13	37.1379	<0.001
parent	0.277498	110	24	6.269529	0.012
life	0.246416	62	16	7.419782	0.006
son	0.222566	19	8	12.22146	<0.001
us	0.21693	80	16	2.384158	0.123
event	0.210042	12	6	12.56353	<0.001
move forward	0.175035	12	5	7.36151	0.007
care	0.166378	34	8	2.431838	0.119

Note: LEMMA_B: base form of element B as it appears in the analysis. COEFF: association coefficient between elements A and B. CE_B and CE_AB: expected counts; CHI2: chi-square statistic (χ^2^); (*p*): *p*-value.

**Table 8:** Associations with the word “Support”.

LEMMA_B	COEFF	CE_B	CE_AB	CHI2	(*p*)
psychological	0.379049	24	10	55.04315	<0.001
figure	0.225189	17	5	16.45652	<0.001
important	0.175075	18	4	8.330094	0.004
search	0.154881	92	8	1.260163	0.262
child	0.145361	235	12	0.918758	0.338

Note: LEMMA_B: base form of element B as it appears in the analysis. COEFF: association coefficient between elements A and B. CE_B and CE_AB: expected counts; CHI2: chi-square statistic (χ^2^); (*p*): *p*-value.

## Data Availability

Interview transcripts are available from the corresponding author upon reasonable request, subject to participant confidentiality constraints. Data are not publicly available due to ethical restrictions. The full interview guide is provided in Appendix A.

## Abbreviations

The following abbreviations are used in this manuscript:

CF	Compassion Fatigue
EOL	End-of-Life
PPC	Pediatric Palliative Care
FCC	Family Centered Care
MBI	Maslach Burnout Inventory
ProQOL	Professional Quality of Life Scale
SICP	Società Italiana di Cure Palliative
ICU	Intensive Care Unit
WHO	World Health Organization
ICD	International Classification of Diseases
JD–R	Job Demands–Resources

## Appendix A. Interview Guide

Structural and organizational correlates of compassion fatigue in pediatric oncology nursing: a qualitative study

Part 1—Sociodemographic Section

Age; gender (male/female/non-binary); educational qualification; years of service; professional role.

Part 2—Semi-Structured Interview Track

The interview was originally administered in Italian. The Italian source text is reported in italics, followed by the English translation used for analysis and reporting. Interview questions were derived from qualitative studies conducted in comparable pediatric palliative care settings. Two pilot interviews were conducted with nurses not included in the final sample to verify the effectiveness of the questions; pilot interviews were not included in the data analysis.

1. Quality of Nursing Care

“Come descriveresti la qualità dell’assistenza infermieristica nel bambino oncoematologico terminale?”

How would you describe the quality of nursing care for the terminally ill oncological/hematological child?

(a) Quali sono i bisogni di cui necessita il bambino?—What needs does the child have?

(b) Quali strategie attui nella gestione?—What strategies do you use in managing care?

(c) Episodi/esperienze/esempi—Episodes/experiences/examples

2. Achievement of Maximum Health

“Come arrivare al raggiungimento massimo di salute possibile?”

How can the maximum achievable level of health be reached?

(a) Quali interventi si possono realizzare?—What interventions can be implemented?

(b) Cosa evitare e perché?—What should be avoided, and why?

(c) Episodi/esperienze/esempi—Episodes/experiences/examples

3. Relationship with Parents

“Come si relaziona con i genitori del bambino?”

How do you relate to the child’s parents?

(a) Che tipo di rapporti si instaurano?—What type of relationships develop?

(b) Quali strategie di cura del lutto si possono attuare?—What grief-care strategies can be implemented?

(c) Episodi/esperienze/esempi—Episodes/experiences/examples

(d) C’è un follow-up successivamente alla morte del bambino?—Is there any follow-up after the child’s death?

4. Coping with the Child’s Death

“Come far fronte alla morte di un bambino?”

How do you cope with the death of a child?

(a) Quali sentimenti predominano?—What feelings predominate?

(b) Quali difficoltà si incontrano?—What difficulties are encountered?

(c) Ritiene che la morte di un bambino influenzi la vita lavorativa? (Se sì) in che modo?—Do you feel that a child’s death affects your working life? (If yes) in what way?

5. Impact on Personal and Professional Life

“Ha un impatto sulla vostra vita privata?”

Does it have an impact on your private life?

(a) Quali cambiamenti apporta questo evento?—What changes does this event bring about?

(b) Le è mai capitato di continuare a sentire i genitori del bambino anche dopo la sua morte? Se sì, perché?—Have you ever continued to stay in touch with the child’s parents after his/her death? If so, why?

(c) Quali sentimenti le ha suscitato?—What feelings did this evoke in you?

Mapping of Interview Questions to Core Study Constructs

**Question Area**	**Core Construct(S) Explored**	**Corresponding Theoretical Framework**
1. Quality of nursing care	Perceived adequacy of care; training gap; procedural vs. emotional care	Job resources (JD–R)
2. Achievement of maximum health	Quality-of-life orientation vs. cure orientation; time and staffing constraints; teamwork	Job demands/job resources (JD–R)
3. Relationship with parents	Trust-building with families; grief-care strategies; presence/absence of institutional follow-up	Relational/emotional labor; job resources (JD–R)
4. Coping with the child’s death	Emotional response to death; occupational difficulties; perceived impact on professional functioning	Compassion fatigue; moral distress; job demands (JD–R)
5. Impact on personal and professional life	Spillover into private life; continued contact with bereaved parents; emotional intrusion	Compassion fatigue; emotional labor; psychological well-being (auxiliary lens: Ryff)
Note: This mapping reflects the a priori rationale for question design; the Job Demands–Resources (JD–R) model was the only framework built into the interview guide itself. The dual-process perspective, compassion satisfaction, emotional labor, moral distress, and Ryff’s model of eudaimonic well-being were applied post hoc during interpretation of the resulting themes (see Section 2 and Limitations, Section 5) and are therefore not claimed here as constructs the guide was designed a priori to assess.		
